# Online intervention, ‘MePlusMe’, supporting mood, wellbeing, study skills, and everyday functioning in students in higher education: a protocol for a feasibility study

**DOI:** 10.1186/s40814-015-0029-8

**Published:** 2015-10-08

**Authors:** Marietta Papadatou-Pastou, Rhianna Goozée, Elizabeth A. Barley, Mark Haddad, Patapia Tzotzoli

**Affiliations:** 1 School of Education, Research Centre for Psychophysiology and Education, National and Kapodistrian University of Athens, Athens, Greece; 2 Department of Psychosis Studies, Institute of Psychiatry, King’s College London, London, UK; 3 Florence Nightingale Faculty of Nursing and Midwifery, King’s College London, London, UK; 4 School of Health Sciences, City University London, London, UK; 5 Department of Neuropsychology, Queen’s Hospital, Romford, UK

**Keywords:** Online intervention, Mood, Mental health, Wellbeing, Depression, Anxiety, Study skills, Academic self-efficacy, MePlusMe, Higher education

## Abstract

**Background:**

Psychological and study skill difficulties faced by students in higher education can lead to poor academic performance, sub-optimal mental health, reduced study satisfaction, and drop out from study. At the same time, higher education institutions’ support services are costly, oversubscribed, and struggle to meet demand whilst facing budget reductions. The purpose of the proposed study is to evaluate the acceptability of a new online intervention, MePlusMe, aimed at students in higher education facing mild to moderate psychological and/or study skill difficulties. The study will also assess the feasibility of proposed recruitment and outcome assessment protocols for a future trial of effectiveness. The system supports self-management strategies alongside ongoing monitoring facilitated by a messaging service, as well as featuring a built-in community of student users. It is based on current clinical guidelines for the management of common mental health problems, together with best practice from the educational field.

**Methods/design:**

Two hundred and forty two students will be recruited to a within-subjects, repeated measures study conducted over 8 weeks. Self-report measures of depression and anxiety symptoms, mental wellbeing, academic self-efficacy, and everyday functioning will be collected at baseline, and then at 2, 4, and 8 weeks. During this period, students will have access to the intervention system. UK higher education institutions Bournemouth University and University of Warwick will participate in the study. Data on student satisfaction and engagement will also be collected. Study findings will help to determine the most appropriate primary outcome and the required sample size for a future trial.

**Discussion:**

This study will evaluate the acceptability of an online intervention system for students facing psychological and/or study skill difficulties and will test recruitment procedures and outcome measures for a future trial of effectiveness. The system is designed to be implemented as a stand-alone service or a service complementary to student support services, which is accessible to the majority of students and effective in improving student experience at higher education institutions.

## Background

There is growing concern about the mental health and wellbeing of higher education (HE) students [1–4], not least because of associations with academic performance. Higher education institutions (HEIs) have an obligation to provide support for the mental health and wellbeing of their enrolled students. Demand for student support services (SSS) is increasing [5], which is likely to result in increased waiting times before students receive support. This may increase the risk of their problems escalating, and may lead to negative consequences for both the individual and HEIs. The proportion of HE students who fail to complete their studies has recently risen to more than 22 % in the UK [3]. Recent UK HEI fee increases [6, 7] add further pressure on HEIs to be cost-effective, show quality and value for money, and enhance ‘student experience’—an important influence on the ratings, intake, and income of HEIs.

**Fig. 1:**
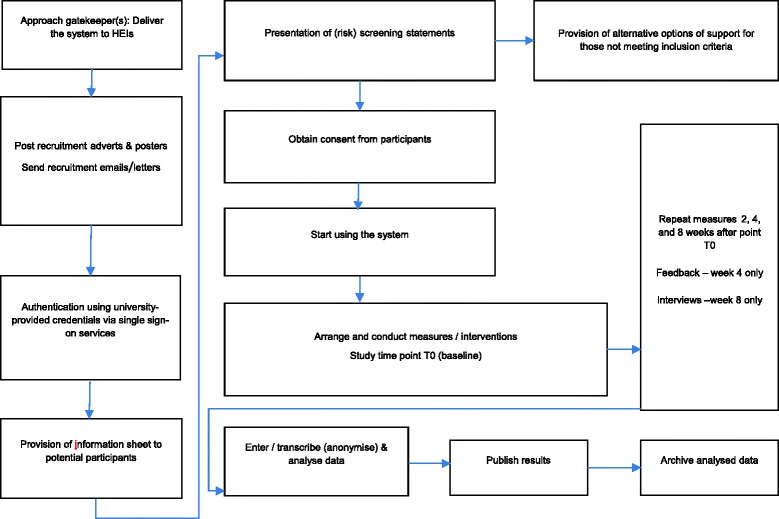
Overview of the study procedure

A number of reports have documented the mental health problems experienced by HE students [2–4, 8–10]. This literature indicates a high prevalence of mental health problems, although there is an absence of well-conducted studies in this area. Responses to a recent electronic survey conducted by the UK National Union of Students showed that 8 % of the students identified as ‘having a mental health problem but not seeking diagnosis’, 2 % identified as ‘currently seeking for a diagnosis’, and 10 % of students identified themselves as having been diagnosed with a mental health problem and believed this diagnosis still applied to them [10]. Alongside this, the Royal College of Psychiatrists [4] noted that 4 % of HE students in the UK seek help from a counsellor for emotional and psychological difficulties each year. Official statistics from HESA suggest that the proportion of students in the UK declaring a mental health difficulty on entry to university has gone up from 0.5 % between 1994 and 1995 to 3.6 % between 2006 and 2007, while numbers have doubled between 2012 and 2013 [6]. A much larger proportion experience psychological or study skill-related difficulties that keep them from achieving their true academic potential and enjoying the university experience to its fullest [4, 9]. This finding is supported by another UK study that found 90.5 % of students surveyed would rank exams or assessments as reasonably stressful or very stressful, while other stressors identified included time management and deadlines (83.3 %), and considering career prospects (75.2 %) [11].

Alongside these challenges, HEIs are facing major cuts to their budgets. In the last UK Governmental Spending Review, cuts of up to 40 % over 4 years were announced [12]. This further challenges the ability of UK HEIs to offer effective student support services. On-site SSS exist to deal with students’ psychological and study skill difficulties, yet limited resources mean that they struggle to cope with the high demand [4, 9]. In some cases, the average waiting time between referral and assessment for counselling is 9 weeks, i.e. more than one academic term [13]. Seeking help via private routes is an alternative option, but usually at a cost unlikely to be affordable for most students. A further concern is that the high demand reported is unlikely to mirror true needs. As many as 30 % of students would not feel comfortable to report their concerns [11], and consequently their needs remain unidentified and thus unmet.

Therefore, alternative means of supporting students should be sought, especially for students who experience mild to moderate difficulties that impact on their studies and student experience and whose needs are not prioritised within HEIs SSS. The Royal College of Psychiatrists [4] has proposed ‘to increase the availability of, and access to, self-help programs such as proprietary or web-based interactive cognitive-behavioural therapy (CBT)’ (p. 20). Online interventions are effective, easy-to-use, low-cost, and can be used anonymously without the potential stigma related to face-to-face treatment [14]. The internet is rapidly expanding as a support tool for psychological problems [15] and as a platform for the delivery of psychological therapies [16, 17]. Treatment of depression using internet-based CBT or problem-solving therapy (PST) has been found to be successful [18, 19], and potentially as effective as face-to-face therapy [19]. A recent systematic review indicated that for many mental health problems, there is little difference between different self-help approaches and whether or not there was guidance and administration by a practitioner [20]. Moreover, internet-based support systems can offer access to psychological and study skill support for a complete cohort of students across each HEI, overcoming the time and resource limitations to which traditional support services are subject.

However, there is limited literature regarding existing internet-based support systems specifically designed for HE students. The few online systems that are on offer to address psychological needs of adults, such as ‘MoodGym’ [21], ‘e-couch’ [22], and ‘Living Life to the Full’ [23], are designed for the general population. A system currently on offer by some HEI for the support of their students is Computer Aided Lifestyle Management (CALM), but there is very limited material describing its development or investigating its feasibility, effectiveness, or student satisfaction [24]. Another online psychological treatment is the ‘SilverCloud Health’ platform [25], which provides help and support packages. Current programmes available target symptoms of depression, stress, anxiety, and eating disorders. However, neither ‘SilverCloud’ nor ‘CALM’ address study skill problems. MePlusMe will fill this gap.

### MePlusMe

Recently, iConcipio has developed an online multimedia intervention called MePlusMe, which meets the need for a system addressing mild to moderate psychological and/or study skill difficulties of HE students. It is an easy-to-use system offering two different routes, a symptoms-route (‘Questionnaire’) or a techniques-route (‘Library’), to access support techniques that match differing styles and needs, ensuring maximum flexibility and utility of the system. Students can practice these techniques in their own time and space, as and when needed, whilst using a rating system to monitor their progress. This ongoing self-management aims to promote students’ personal effectiveness in addressing internal challenges and environmental demands. The self-monitoring process is further supported by a messaging service, in the form of motivational reminders to the user’s inbox within the system, to their personal email accounts, and, if they choose to, to their personal mobile phone. These messages encourage them to return to the system to practise the techniques and complete the post-intervention measures. Furthermore, a social network where students can post their thoughts on the ‘Thought Wall’ and other students have the option to ‘like’ or ‘share’ these thoughts in other social media (Facebook and Twitter) is present, allowing students to interact with each other anonymously for mutual support.^1^ This built-in community of students acts as an additional support resource, normalising the students’ experience and giving them a feeling of belonging and fitting into a group. The social network is monitored and regulated by expert facilitators. Moreover, a number of filters are embedded within the system to ensure that only students with mild to moderate difficulties are using it. Where applicable, those with more significant difficulties are referred to more appropriate services, such as their HEI student support mechanisms or mainstream helplines and help centres.

Students who want guided help can start using the system by completing the Questionnaire (symptoms-route). This interactive questionnaire follows a specific taxonomy to identify students’ psychological difficulties, focusing on anxiety and depression symptom. Additionally, symptoms of anxiety and depression have a high comorbidity. This was taken into account in the development of the initial Questionnaire, which differentiates between students who experience only anxiety symptoms, only depression symptoms, or a combination of both. The Questionnaire was designed to differentiate between the different types of presenting problem that were identified during development of the system as most prevalent in the target group (students) and for which there was clear evidence for the effect of this mode of supported self-help intervention.

The version of the Questionnaire in the described trial differentiates between three broad types of presenting problem, involving either predominant features of anxiety, or of depression, or mixed anxiety and depression features. The questionnaire statements have been adapted from established tools, including clinical questionnaires (Hospital Anxiety and Depression Scale [HADS] [26]; 7-item Generalised Anxiety Disorder Scale [GAD-7] [27]; Patient Health Questionnaire [PHQ-9] [28]) and a formal interview (Mini International Neuropsychiatric Interview [MINI] [29]). The HADS, GAD-7, and MINI informed the choice of anxiety questions. The HADS, PHQ-9, and MINI informed the choice of depression questions. iConcipio undertook extensive pilot testing (*n* = 491) that showed these questionnaire items to be acceptable and to effectively provide initial filtering of presenting problems (Tzotzoli, personal communication). However, the Questionnaire is designed in such a way that it is expandable. Future launches of the system may include more questions and thus may cover a wider array of psychological difficulties. Upon completion of the Questionnaire, a package with techniques is suggested based on the best-fit intervention tailored to students’ identified needs.

An alternative route that can be followed by students is via the Library (techniques-route). Here, students can freely browse all the available psychological and educational techniques and create a personalised package to help them improve in the areas of personal effectiveness on which they choose to focus. Both routes lead to a personal space called ‘My Place’, where students will find their package of techniques, either the package assembled for them as determined by their ‘Questionnaire’ answers or the package that they have assembled themselves in the ‘Library’.

The psychological techniques are derived from CBT. There is strong evidence supporting the effectiveness of CBT in addressing a range of emotional difficulties and associated behaviours [30–33]. Techniques considered by the expert clinical team to most likely be acceptable, feasible, and effective in the target HE population were selected for inclusion in this intervention. The educational techniques consist of the most up-to-date practical skills, extensively tested and shown to improve students’ performance [34]. They aim to help students focus their efforts better and develop successful study skills and strategies.

The techniques are presented in the form of animated videos which demonstrate evidence-based psychological and/or study skill techniques. Videos are a pioneering media for communicating evidence-based techniques to non-expert audiences, as research shows that multimedia aids learning by engaging both verbal and visual information-processing channels [35, 36].

The system has a ‘bottom-up’ design, following a symptoms- and a techniques-driven approach, to avoid the potential stigma that may be associated with applying labels to difficulties. It aims to achieve more effective and immediate results by focusing on how to ‘cope’ better with the student’s current challenges. The language, visual appearance, and feel of the system are also chosen carefully to address its target group. Overall, this online intervention is based on current scientific knowledge and best practice, and provides a mosaic comprising implicit (e.g. nudge theory, [37]) and explicit (e.g. relaxation) state-of-the-art psychological and educational strategies (e.g. how to prepare for exams).

### Development

The development of MePlusMe has followed the Medical Research Council (MRC) guidelines for developing complex interventions [38]. The system has been developed in three stages, in collaboration with both students and HE providers. Initially, we conducted two market research projects. The first targeted counsellors and psychologists working in HEI SSS, using a semi-structured interview to collect information regarding the operation of existing services, and their common practices, needs, and challenges. The second project was conducted with HE students via an online survey, which will be detailed in a future publication (Goozée, Papadatou-Pastou, Barley, Haddad, & Tzotzoli: Survey to inform the development of an online support system for higher education students: a brief report, sumitted). This research sheds light on the most common difficulties that students face at universities, their opinions of online support systems, and what features they believe make such systems useful.

Recently, a proof of concept study was completed with the participation of five UK HEIs (King’s College London, University of Edinburgh, University of Roehampton, Bournemouth University, and University of Warwick; *n* = 873 students) to ensure that the development of MePlusMe is acceptable and reflects end-users’ needs [Touloumakou, Goozée, Papadatou-Pastou, Barley, Haddad, & Tzotzoli: Elearning support system for HE students with psychological and study skill difficulties: proof of concept study,” submitted]. During this study, uncertainties that were identified in the development of the system were examined, and feedback regarding the system’s acceptability and feasibility was obtained. Encouragingly, preliminary data showed that students like and need such a system.

In addition, an in-depth discussion with executives from HEIs took place following testing to gather their thoughts and opinions. This feedback alongside findings from the proof of concept study allowed refinement of the initial design. As an example, wording and graphics were altered to better appeal to an older student group, as well as younger students.

Moreover, the system is being continually developed in collaboration with two advisory boards. Firstly, the universities’ advisory board (UAB) ensures that MePlusMe addresses the requirements of HEIs services, as well as student needs. Secondly, iConcipio’s research and clinical team, consisting of senior academic and clinical psychologists from various HEIs and NHS hospitals, ensures that MePlusMe adheres to the best psychological practice, and that all research projects follow relevant guidelines.

### Aims of study

Following the activities outlined above, a full-scale feasibility study is now warranted. The study will commence in spring 2016 and recruitment of HEIs started in spring 2014. The study will evaluate the feasibility, acceptability, and potential effects of MePlusMe. It will specifically evaluate potential effects on students’ mood (symptoms of anxiety and depression), mental wellbeing, study skills, and everyday functioning, and their engagement and satisfaction with the system. This paper describes the protocol for the feasibility study. This is a crucial step, as its intended outcomes will inform a randomised controlled trial (RCT), leading to a wide-scale incorporation of the system within HEI SSS.

## Methods/design

### Setting and participants

Two UK HEIs that initially participated in our preliminary audit work (Bournemouth University and University of Warwick) will participate in the present study and will be given online access to MePlusMe.

All students enrolled at these HEIs will be eligible to be screened for inclusion. Any student with self-reported mild to moderate psychological and/or academic-related difficulties can participate in the study. Students with more significant difficulties will be excluded via a screening process within the system. All students will be presented with screening statements, including items on engagement in risky behaviours such as self-harm, substance abuse, and physical harm to others, as well as unusual sensory experiences or beliefs. Those who feel that any of the statements apply to them will be discouraged from proceeding to use the system, and will be directed to more appropriate sources of support for their difficulties.

### Study design

This feasibility study will use a within-subjects, repeated measures design to assess changes in mood (symptoms of depression and anxiety), mental wellbeing, study skills, and student engagement and satisfaction. These data will be used to evaluate potential primary outcome measures and inform the sample size for a future RCT. Figure 1 provides an overview of the study procedure. Ethical approval covering all sites was granted by Kings College London (KCL) College Research Ethics Committee (CREC), Psychiatry, Nursing and Midwifery Research Ethics Subcommittee (PNM RESC; Research Ethics Reference Number: PNM/13/14-125). This study will commence in spring 2016 and recruitment of HEIs started in spring 2014.

### Recruitment and consent

The participating HEIs will receive support from iConcipio to promote the study to their enrolled students via their usual communicating channels (social media, website, leaflets, and circular emails). Moreover, if the HEIs agree, SSS will be able to offer this system to students who are currently on their waiting list.

Students who are willing to participate in the study will log-in to the system and will be authenticated using university-provided credentials via single sign-on (SSO) services (e.g. Shibboleth, OAuth, LDAP lookup).^2^ They will then be directed to the information sheet. If they decide to proceed, they will access a screen listing risk statements (e.g. self-harm, substance abuse, physical harm to others, unusual sensory experiences or beliefs). At this stage, the students will not be asked to declare whether they identify with any of the statements, but they will be prompted to select either (i) the ‘proceed’ button if none of the statements applies to them or (ii) the ‘next’ button in case they need further guidance, which will lead them to a screen referring them to more appropriate services, such as their HEI student support mechanisms or mainstream helplines and help centres. If eligible (i.e. if they choose the ‘proceed’ button), the students will have access to a consent form, which they will need to sign in order to be able to register their details and use the system.

### Intervention

Following screening, students will decide whether they want to access the Library, directly choosing the techniques they are interested in or whether they want guided help, entering the Questionnaire route to identify their difficulties. Should they choose the Questionnaire route, the students will be presented with the initial screen of the system which poses the question ‘What is your hotspot today?’ allowing each student to come into the questionnaire with whatever they think the presenting problem is. The three entry points are (i) ‘It’s how I am feeling inside’, addressing psychological difficulties, (ii) ‘It’s my studies’, addressing study skills problems, and (iii) ‘It’s outside pressures’. However, the questionnaire is designed in such a way that no matter which entry point the student chooses, they will always be directed to the other points at the end of each section.

Once they choose the techniques they are interested in from the Library or upon completion of the Questionnaire, the student will be asked to rate how much their difficulties are currently affecting their life. They will then have the opportunity to name their package of techniques so that they can later find it in their personal space. At this stage, they will also decide whether they want reminders to encourage them to return to watch the videos and practice the techniques. They will then be guided to My Place where they will have access to the package with the techniques. The student will be asked to rate again at different intervals (2, 4, and 8 weeks from baseline) how much their difficulties are affecting their life after practising these techniques, and their responses will result in a graph indicating their progress over time as a self-monitoring tool. If the students give two consecutive low ratings, they will be prompted by email with information on where to seek further support. Moreover, it is stated in the description of some techniques (e.g. the two psychoeducation techniques of understanding stress and understanding low mood) that if symptoms persist for more than 2 weeks, then students should seek support elsewhere (specific referrals are again provided). Students can make notes of their thoughts regarding the techniques and their experience of them, and are given the option either to keep their thoughts private or to share them within the system’s built-in social network.

### Data collection

The system will collect data on age, gender, ethnicity (based on the UK Census 2011 categories), student status (home, EU, international), and course of study from the participating students.

### Outcome measures

#### Mood and wellbeing

Upon submission of the consent form, students will complete validated clinical measures, namely the PHQ-9 [28], a widely used 9-item self-report measure of depression symptoms, and the GAD-7 [27], a 7-item scale commonly used in clinical and research practice to assess for current symptoms of anxiety and their severity. The Edinburgh-Warwick Mental Wellbeing scale will be used to assess positive mental health (mental wellbeing). This is a 14-item scale initially validated using student populations and additionally used in national survey studies.

#### Everyday functioning

To measure everyday functioning, participants will be asked to respond to the question ‘How well are you managing now in your daily life?’ using a 5-point Likert scale, ranging from ‘extremely well’ to ‘not at all’.

#### Study skills

Self-efficacy will be assessed using an Academic Self-Efficacy scale adapted with the permission of its authors [39].

#### User satisfaction and engagement

This information will be gathered via a feedback form completed by the users, which consists ofboth open-ended and Likert-scale questions regarding the usefulness of the techniques, whether the users like how the system looks and works, fidelity (how often they used and returned to the system and whether they would prefer alternative methods of support), personal self-efficacy (whether they feel in control of their problems since using the system), and whether they presented to student support services whilst or after using the system. The system will also automatically record the following information: the average duration of MePlusMe visits, the total number of visits over the study period, the number and nature of missing answers (i.e. compliance of students to the complete filling-in of the forms), the number of students signed up for the study, and log-in patterns.

#### Acceptability of intervention

A semi-structured interview will take place face-to-face with between 15 and 20 randomly selected participants who will have expressed an interest in providing qualitative information on their experience of using MePlusMe. For inclusion in the interviews, students will have to have used the system for two full months and completed all measurements. We will also recruit students who dropped out before the full 2 months to assess their reasons for doing so. The interview will last approximately 20 min, will be recorded with consent, and will be transcribed verbatim. It will explore a number of issues, such as the elements of the system that the participants liked or did not like (quality of implementation), the ways in which they felt that the system helped them or not, how often they used the system, whether they would have prefered to consult a counsellor or to have had access to alternative methods of support instead (fidelity), and what barriers might stop them from using the system in the future.

### Assessments

Students will be asked to complete the measures of mood (symptoms of depression and anxiety), mental wellbeing, functioning, and study skills at baseline (T0) prior to using the intervention but after giving consent, and then again at 2 weeks (T1), 4 weeks (T2), and 8 weeks (T3) from baseline. The satisfaction feedback form will be sent to participants at week 4 (T2). By collecting information at this stage, we hope that the feedback will be more reliable as students will still be practising the techniques that we wish them to appraise. Finally, after 2 months, a semi-structured interview will take place with the subsample of participants (see Table 1).

**Table 1:** Overview of outcome measures

		Time of measurement			
Instrument	Aim	T0 baseline (pre-testing)	T1 post-test (2 weeks)	T2 follow-up (4 weeks)	Τ3 follow-up (8 weeks)
PHQ-9	Symptoms of depression	x	x	x	x
GAD-7	Symptoms of anxiety	x	x	x	x
Edinburgh-Warwick Mental Wellbeing scale	Positive mental health (mental wellbeing)	x	x	x	x
Academic self-efficacy	Academic self-efficacy	x	x	x	x
VAS	Everyday functioning	x	x	x	x
Feedback form	Engagement and satisfaction of the end-users			x	
Interview (15–20 participants)	Qualitative assessment of the experience of using the system				x
Intervention delivery	Patterns of use of the system (e.g. duration of use, total number of visits)	x	x	x	x

### Statistical methods

#### Sample size—power calculation

This is primarily a feasibility study, so hypothesis testing of clinical outcomes is not a key part of the design. Nonetheless, to assist determination of the appropriate sample size for a definitive evaluation of this intervention, and to provide indicative values for outcome measures and change scores, a sample size calculation was conducted using the mean depression scores, standard deviation values, and change scores obtained from the evaluation of an internet depression package and comparable face-to-face psychological interventions [40, 41]. This indicates that for a paired sample *t* test, where power = 0.95 and *α* = 0.05, a total sample size of 47 is sufficient to identify differences in depression rating scores, within the range of similar published studies. For a statistical evaluation of proportional change involving a difference in caseness following intervention to 20 % from a pre-intervention case prevalence of 30 %, a total sample size of 186 will be required (where power = 0.9; *α* = 0.05). Using the more conservative estimate (186), and inflating our required number by 30 % (56) to allow for dropout, indicates that 242 participants should be recruited. Given that 873 students participated in iConcipio’s preliminary audit work, it is reasonable to expect that it will be possible to recruit a sufficiently sized sample for the feasibility study.

#### Data analysis

This being a feasibility study, descriptive findings concerning recruitment numbers, completions, drop-outs, and summary estimates of outcome measures at baseline and follow-up focussing on the dropout rate at each time point will be explored. We will also conduct exploratory analyses of the pre-post effect of the intervention using the data from participants who have completed the programme. To examine differences before and after using MePlusMe, we will conduct a 4 × 4 repeated measures ANOVA with time (four time-points) and the four assessments (PHQ-9, GAD-7, Edinburgh-Warwick Mental Wellbeing scale, Academic Self-Efficacy scale, and VAS ratings) as the within-subject variables. Effect sizes will be presented as Cohen’s *d.* This will enable determination of the outcome measure mean, standard deviation and variance, and change, which will be essential for designing an appropriately powered trial.

Qualitative data from the feedback form will be analysed using content analysis. Interviews and analyses will be performed concurrently using the principles of constant comparison [42] and thematic analysis [43]. At least two researchers will independently code the first interview and agree on descriptive codes. These codes, and where appropriate further new codes, will be applied to subsequent transcripts. Consistency in coding will be checked within the research team. Descriptive codes will be collated into themes and a preliminary explanatory framework devised. This will be used as the basis for coding and for informing future interviews. The robustness of themes will be tested by examining differences and similarities between coded data.

## Discussion

This study protocol is designed to test the feasibility, acceptability, and potential effects of a new online multimedia intervention, MePlusMe, which addresses mild to moderate psychological difficulties, as well as study skill difficulties experienced by HE students. The novelty of the system warrants an initial study to provide feasibility data. These data will provide valuable information regarding feasibility of recruitment strategies (including sample size) and the proposed outcome measures, alongside the extent of change between pre- and post- intervention ratings (mood, wellbeing, academic self-efficacy, and everyday functioning) and acceptability of the intervention, informing the implementation of future randomised trials assessing effectiveness.

MePlusMe provides students with an immediate personalised resource for support, which they are able to access in privacy from wherever they are, as often as they want, and at any time. In this way, they can preserve anonymity whilst developing their personal effectiveness and coping skills. The system offers a holistic and inclusive service by reaching out to the majority of students, even those who would not normally present to SSS for their difficulties. In addition, the system is designed in such a way that it can address a variety of needs as they change over time, and provides instant, tailor-made support. A multimedia format of the provided support renders the experience easy, fun, and thus more accessible and user-friendly. The system reinforces and empowers students to take responsibility for their wellbeing, and this in itself strengthens their self-efficacy and self-esteem.

In addition to the support that the students can enjoy by using the system, HEIs can also benefit from introducing MePlusMe to their students. It can represent a complementary, high-quality service alongside their existing support mechanisms. For example, personal tutors, untrained in counselling, may use the system as a first option for students experiencing difficulties. Moreover, counsellors can refer students with milder difficulties to the system, allowing them to concentrate on those especially in need of professional support. As such, MePlusMe can act as a filtering portal for the HEI SSS, and assist them to optimise their resources, whilst minimising their running costs. MePlusMe can further be used by students who are on a waiting list to receive support from SSS, ensuring that the students have some form of support in the meantime. After completing their sessions at the SSS (usually four or five sessions per student), the students can use MePlusMe as a complementary back-up support system. Whilst it can complement current services, the system can also act as a stand-alone resource, thus enriching HEIs offerings to their students. Α current limitation of MePlusMe is that the benefits of face-to-face psychological and study skill support cannot be provided. Future versions of the system are likely to include more direct modes of support by specialised professionals, either through emails, video-conferencing, or via offline face-to-face treatment.

The system is designed in such a way that it ensures that HEIs can attract and support students from different ethnic backgrounds, and help them fulfil their obligations to international students. The user data analytics provided by MePlusMe can offer HEIs access to their students’ mental health and academic needs, which can further assist them to identify how existing services can be resourced to reach higher efficiency. Therefore, MePlusMe can be part of the restructuring agenda for HEIs SSS, assisting them to promote themselves as a ‘caring university’, more effectively meet their pastoral role, increase course completion rates, reduce financial losses from drop-outs, optimise their resources, enhance students’ experience and satisfaction, improve their rankings, and increase their annual intake of students. This will aid their accountability to stakeholders on resources spent, and assist them to retain their funding and remain financially positive.

Overall, MePlusMe addresses common challenges faced by students by offering pragmatic coping mechanisms that de-stigmatise difficulties. Informed by current evidence-based psychological practice, it emphasises prevention rather than treatment. In addition, it represents a holistic solution for student support, which could minimise costs, while potentially offering a high-quality service. It is designed to reach out to the majority of students who do not require formal services, but still have difficulties that need to be addressed. In the current version, the psychological problem types addressed are anxiety symptoms, depression symptoms, and mixed anxiety and depression symptoms. Yet, the questionnaire used is designed in such a way that it is expandable. Thus, by adding more questions, it could further discriminate specific anxiety features. In future versions, features of conditions, such as social anxiety and seasonal affective disorder (SAD), will be considered for inclusion. Accordingly, new and appropriate techniques will be included to address these newly introduced conditions.

## Study status

The study will commence in spring 2016 and recruitment of HEIs started in spring 2014. The study will evaluate the feasibility, acceptability and potential effects of the first online, tailored intervention to address mild to moderate psychological and study skills difficulties in HE students.

## Abbreviations

CBT: cognitive behavioural therapy
CREC: College Research Ethics Committee
GAD: generalised anxiety disorder
HADS: Hospital Anxiety and Depression Scale
HE: higher education
HEI: higher education institutions
KCL: Kings College London
MINI: Mini International Neuropsychiatric Interview
PHQ: Patient Health Questionnaire
PNM RESC: Psychiatry, Nursing and Midwifery Research Ethics Subcommittee
RCT: randomised controlled trial
SAD: seasonal affective disorder
SSS: student support services
UAB: universities’ advisory board
VAS: visual analogue scale

## Footnotes

1 When students post on the ‘Thought Wall’, their thoughts appear under their preferred username; thus, their anonymity is safeguarded. When they share their own thoughts or the thoughts of others on their Facebook or Twitter accounts, again, these thoughts are linked to the username originally used to post the thoughts. Therefore, unless the Facebook friends or the Twitter followers of a student know his/her unique username, they will not be able to link the thought(s) back to the student.

2 Using SSO services means that students will identify themselves away from the system. The logon process is performed on each institution’s server, directly between the student and the institution. Once the students provide their credentials there, the institution’s server forwards to our system a unique identifier for this user. The form of this unique identifier (numbers, letters, etc.) is wholly dependent on how the institution configures their side of the SSO process. In this way, MePlusMe does not have access to the student’s actual credentials (log-in or password). For the purposes of the feasibility study, we will request more information from the institutions on the backend (e.g. student’s department and year of study). This data will be then used to pre-fill the registration form for the study, once the SSO process is complete and the student is back on our system. The student can then edit this information at will. From then on, the data is handled confidentially: (a) it is immediately stored on a separate database from the rest of the system; (b) it is encrypted with a key only the researchers have access to; (c) the database is configured to be read-only for the system, so the system can only add records and not read them; (d) read access is only provided to the researchers; and (e) the data is not logged. The above information will be available (a) during the log-in/registration process and (b) under the section ‘Frequently Asked Questions’ on the Menu.

## References

[1] Rana R, Smith E, Walkling J. Degrees of disturbance: the new agenda; the Impact of Increasing Levels of Psychological Disturbance Amongst Students in Higher Education. Rugby, England: Association for University and College Counselling, 1999.

[2] Committee of Vice-Chancelors and Principals (CVCP) (2000). Guidelines on student mental health policies and procedures for higher education.

[3] Higher Education Statistics Agency (2011). “UK domiciled HE students by level of study, gender, mode of study, year of study and disability status 2009/10,”.

[4] Royal College of Psychiatrists, “The Mental Health of Students in Higher Education (Council Report CR166),” 2011. [Online] Available: http://www.ox.ac.uk/sites/files/oxford/field/field_document/Royal%20College%20of%20Psychiatrists%20Metal%20Health%20of%20Students%20in%20HE_1.pdf. [Accessed 2-Oct-2015].

[5] Watkins DC, Hunt JB, Eisenberg D (2012). Increased demand for mental health services on college campuses: perspectives from administrators. Qual Soc Work.

[6] Higher Education Statistics Agency. “Free Online Statistics - Students & qualifiers,”. 2013 [Online]. Available: https://www.hesa.ac.uk/index.php?option=com_content&view=article&id=1897 [Accessed: 2-Oct-2015].

[7] Department for Business Innovation and Skills (BIS) (2011). Higher Education: students at the heart of the system, no. June.

[8] Universities UK (2002). Reducing the risk of student suicide: issues and responses for higher education institutions.

[9] Royal College of Psychiatrists (2003). “The mental health of students in higher education (Council Report C112),”.

[10] National Union of Students (2013). “Mental distress survey overview,”.

[11] National Union of Students (2010). “Silently stressed: a survey into student mental wellbeing,”.

[12] HM Treasury (2010). “Spending review,”.

[13] Connell J, Barkham M, Mellor-Clark J (2007). CORE-OM mental health norms of students attending university counselling services benchmarked against an age-matched primary care sample. Br J Guid Counc.

[14] Corrigan P (2004). How stigma interferes with mental health care. Am Psychol.

[15] van der Houwen K, Schut H, van den Bout J, Stroebe M, Stroebe W (2010). The efficacy of a brief internet-based self-help intervention for the bereaved. Behav Res Ther.

[16] Carlbring P, Maurin L, Törngren C, Linna E, Eriksson T, Sparthan E (2011). Individually-tailored, internet-based treatment for anxiety disorders: a randomized controlled trial. Behav Res Ther.

[17] So M, Yamaguchi S, Hashimoto S, Sado M, Furukawa TA, McCrone P (2013). Is computerised CBT really helpful for adult depression?—a meta-analytic re-evaluation of CCBT for adult depression in terms of clinical implementation and methodological validity. BMC Psychiatry.

[18] Spek V, Cuijpers P, Nyklícek I, Riper H, Keyzer J, Pop V (2007). Internet-based cognitive behaviour therapy for symptoms of depression and anxiety: a meta-analysis. Psychol Med.

[19] Cuijpers P, Donker T, van Straten A, Li J, Andersson G (2010). Is guided self-help as effective as face-to-face psychotherapy for depression and anxiety disorders? A systematic review and meta-analysis of comparative outcome studies. Psychol Med.

[20] Farrand P, Woodford J (2013). Impact of support on the effectiveness of written cognitive behavioural self-help: a systematic review and meta-analysis of randomised controlled trials. Clin Psychol Rev.

[21] Christensen H, Griffiths K, Groves C (2004). MoodGYM training program: clinicians manual.

[22] Griffiths KM, Crisp D, Christensen H, Mackinnon AJ, Bennett K (2010). The ANU WellBeing study: a protocol for a quasi-factorial randomised controlled trial of the effectiveness of an Internet support group and an automated Internet intervention for depression. BMC Psychiatry.

[23] Williams C, Wilson P, Morrison J, Mcmahon A, Andrew W, Allan L (2013). Guided self-help cognitive behavioural therapy for depression in primary care: a randomised controlled trial. PLoS One.

[24] Davis-McCabe C, Winthrop A (2010). Computerised CBT: university students experiences of using an online self-help programme. Couns Psychol Rev.

[25] Richards D, Timulak L, Doherty G, Sharry J, Colla A, Joyce C (2014). Internet-delivered treatment: its potential as a low-intensity community intervention for adults with symptoms of depression: protocol for a randomized controlled trial. BMC Psychiatry.

[26] Zigmond AS, Snaith RP (1983). The hospital anxiety and depression scale. Acta Psychiatr Scand.

[27] Spitzer RL, Kroenke K, Williams JBW, Lo B (2006). A brief measure for assessing generalized anxiety disorder. Arch Intern Med.

[28] Spitzer RL, Williams JBW, Kroenke K, Hornyak R, Mcmurray J, Questionnaire H (2000). Validity and utility of the PRIME-MD Patient Health Questionnaire in assessment of 3000 obstetric-gynecologic patients: The PRIME-MD Patient Health Questionnaire Obstetrics-Gynecology Study. Am J Obstet Gynecol.

[29] Sheehan D, Lecrubier Y, Sheehan K, Amorim P, Janavs J, Weiller E (1998). The Mini-International Neuropsychiatric Interview (MINI): the development and validation of a structured diagnostic psychiatric interview for DSM-IV and ICD-10. J Clin Psychiatry.

[30] Hunot V, Churchill R, Teixeira V, De Lima M (2007). Psychological therapies for generalised anxiety disorder. Cochrane Database Syst Rev.

[31] Sheldon B (2011). Cognitive-behavioural therapy: research and practice in health and social care.

[32] Dimauro J, Domingues J, Fernandez G, Tolin DF (2013). Long-term effectiveness of CBT for anxiety disorders in an adult outpatient clinic sample: a follow-up study. Behav Res Ther.

[33] Stikkelbroek Y, Bodden DHM, Dekovi M, Van Baar AL (2013). Effectiveness and cost effectiveness of cognitive behavioral therapy (CBT) in clinically depressed adolescents: individual CBT versus treatment as usual (TAU). BMC Psychiatry.

[34] Cottrell S (2008). The study skills handbook (Palgrave study skills).

[35] Mayer F (2001). Multimedia learning.

[36] Mayer RE (2003). The promise of multimedia learning: using the same instructional design methods across different media. Learn Instr.

[37] Sunstein CR, Thaler RH. Nudge: Improving Decisions About Health, Wealth and Happiness. UK: Penguin, 2012

[38] Craig P, Dieppe P, Macintyre S, Health P, Unit S, Michie S (2008). Developing and evaluating complex interventions: the new Medical Research Council guidance. Br Med J.

[39] Tennant R, Hiller L, Fishwick R, Platt S, Joseph S, Weich S (2007). The Warwick-Edinburgh Mental Well-being Scale (WEMWBS): development and UK validation. Health Qual Life Outcomes.

[40] Zajacova A, Lynch SM, Espenshade TJ (2005). Self-efficacy, stress, and academic success in College. Res High Educ.

[41] Christensen H, Griffiths KM, Jorm AF (2004). Primary care delivering interventions for depression by using the internet: randomised controlled trial. Br Med J.

[42] Clark D, Layard R, Smithies R, Richards D, Suckling R, Wright B (2009). Improving access to psychological therapy: initial evaluation of two UK demonstration sites. Behav Res Ther.

[43] Glaser BG (1978). Theoretical sensitivy: advances in the methodology of grounded theory.

[44] Braun V, Clarke V (2006). Using thematic analysis in psychology. Qual Res Psychol.

